# Are the normal values of thyroid gland in children fulfilling the role attributed to them?

**DOI:** 10.1186/1756-6614-8-S1-A27

**Published:** 2015-06-22

**Authors:** Arkadiusz Zygmunt

**Affiliations:** 1 Department of Endocrinology and Metabolic Diseases, Polish Mother’s Memorial Research Institute, The Medical University of Lodz, Lodz, Poland

## 

It has always been very difficult to precisely define a goitre. The borderline cases, when a distinction between healthy and abnormal thyroid has to be made, are especially problematic.

Imperfection of goitre classifications is connected with high variability of thyroid palpation, both interobserver and intraobserver – this variability increases with diminishing thyroid volume and decreasing goitre incidence.

Application of ultrasound examination of the thyroid has significantly decreased variabitity of obtained data, however, repeatability of this examination also depends on many factors, including the thyroid volume, position in which the assessment is being performed, width of the probe, as well as experience and number of the researchers.

Before final analysis, results thus obtained are interpreted by comparison of the measured thyroid volume to the value considered as borderline.

For years, the borderline values, which could be universally used in such evaluations, have been searched for. However, presented reference values were very often disappointing as they proved to be either too restrictive or too liberal. This is one of the reasons why assessment of goiter incidence based even upon ultrasound examination has been losing its significance, giving way to assessment of ioduria, which seems to be more objective evaluation of iodine intake in the examined population.

Having analyzed the suggestions for ultrasound reference ranges in children we came to the conclusion that their inadequacy resulted from erroneous assumptions, which had been made during their development.

## Examined population

A question arises whether reference ranges developed for children living in a region with adequate iodine intake can be universally used for classification of children residing in a different area?

No, this could only be possible if the thyroid size depended merely on the iodine intake. Although it is the iodine deficiency that is the most frequent cause of goiter, other factors can also influence the size of the thyroid. They include goitrogenic agents, naturally occurring in the environment (flavonoids and humus substances deriving from organic debris in the soil), as well as factors connected with pollution. Many inorganic (like thiocyanates, chlorides, nitrates) and organic (like phenols, hydrocarbons, phthalic acid esters) compounds have goitrogenic activity. Diet of the examined population is also important, as long-term consumption of vegetables from *Cruciferae sp.* or poorly cleaned cassava, which contains large quantities of glycosides releasing cyanides metabolized to thiocyanates, can result in significant enlargement of the thyroid gland. On the other hand, consumption of large amounts of saltwater fish or algae provides large, often supra-physiological supply of iodine. A large amount of iodine in ingested food does not always correlate with its high absorption by an organism. Although erythrosine, widely used in food industry as a colouring agent (e.g. in cereals), contains large amounts of iodine, its bioavailability for the human body is low.

When analyzing the results, one should also consider the genetic predisposition of the examined population (its ethnic background) as well as the incidence of autoimmune disorders, which might influence thyroid size too.

The assumption that the size of the thyroid depends only on iodine supply leads to errors, which must be realized when using reference ranges developed so.

## Metrical and anthropometric data

The reference ranges have the character of discrete/non-continuous (grouped) data. Depending on the age or BSA (body surface area) value, the thyroid size of an examined child is compared to the reference group. Groups’ ranges are quite wide and equal: for age – 8.3%-16.7% of the lower value of the range, for BSA – 6.25%-14.3%. Therefore, errors resulting from rounding, can be as high as 8.35% for children in the 6-year-old group and 7.15% in the group of children with BSA of 0.7 m^2^ (these are the youngest and smallest children, in whom the thyroid size is the smallest, so they constitute the group with the highest intra- and interobserver variability).

There is no doubt how to compare the obtained results with the reference ranges based on BSA (given reference value constitutes the middle of the range), however, interpretation of the result obtained on the basis of the age may encounter difficulties.

Age should be determined based on the date of the examination and the date of birth. However, frequently it is defined by the age declared by the parents in the questionnaire, which leads to an error resulting from rounding the real data.

Moreover, ages given in the reference values might be interpreted in two ways – as the middle of the range (like BSA) – in such situations children aged 11.5-12.49 would fit in the 12-year-old group or the inclusion in the 12-year-old group is possible only when the child turns 12 years (an 11.5-year-old child is still in the 11-year-old group).

One should also be aware of the phenomenon of acceleration, as it makes it difficult to compare data from different time points, as well as in the populations inhabiting different latitudes.

Regardless of the different interpretations of the data, it should be remembered that the result obtained based on reference ranges set for the groups, contains an error resulting from rounding (conversion of continuous data into discrete data).

## Variability in iodine intake

Iodine supply in the population is not a constant value and it may change over time. The consumption of iodine in the society is influenced not only by iodine prophylaxis, but also by education policy, aimed at presenting the consequences of iodine deficiency and the necessity of prevention of deficiency of this element in the surrounding environment. The integration processes between countries and more and more expanding free trade and movement of people affect the iodine intake in the population, as well. Foodstuffs (especially imported by private persons) do not necessarily have to meet the standards of the iodine content set by a given country. Models of iodine prophylaxis differ among particular countries – they do not need to be based of obligatory use of iodized salt, like in Poland. Moreover, in recent years a lot of effort has been made to decrease salt intake, which is still the most common carrier for iodine. These are all constant processes, changing the iodine supply in a population. There are examples of countries in which iodine deficiency problems, once solved by introducing preventive measures, reappeared.

Once developed, standards may become outdated over time.

## Conclusions

During the analysis of the results one should use as little rounding and generalizations as possible, because these are subject to error. The data should be processed as little as possible, they should be analyzed as continuous data, and not discrete. Acquiring them should be burdened with the least intra- and interobserver variability.

The analysis should be based on a comparison of the thyroid gland to the parts of the body (like thumb phalanx or BSA) instead of age, due to high variability during the growth period.

Analysis of thyroid volume to BSA (V/BSA) is in our opinion the best estimation of the size of the thyroid gland in the study population. Potential error is only burdened with the error resulting from the measurements (intra- and interobserver variability), like ioduria level determination.

